# Symptomatic Junctional Bradycardia Due to Untreated Hypothyroidism After Beta-Blocker Discontinuation: A Case Report

**DOI:** 10.7759/cureus.40605

**Published:** 2023-06-18

**Authors:** Osman Rahimi, Kevin Lee, Kachon Lei, Sandhya Wahi-Gururaj, Arjun V Gururaj

**Affiliations:** 1 Internal Medicine, Kirk Kerkorian School of Medicine at the University of Nevada, Las Vegas, Las Vegas, USA; 2 Cardiology, Kirk Kerkorian School of Medicine at the University of Nevada, Las Vegas, Las Vegas, USA; 3 Cardiology, University Medical Center, Las Vegas, USA

**Keywords:** paroxysmal atrial fibrillation, thyroid supplementation, hypothyroidism and bradycardia, beta-blocker side effects, junctional bradycardia

## Abstract

Hypothyroidism is known to cause bradycardia, but there are no direct comparisons of the level of thyroid-stimulating hormone (TSH) to heart rate (HR) to assist in therapeutic hormonal management. This case presents a patient who developed symptomatic junctional bradycardia and underwent serial TSH testing to attempt to improve her HR while minimizing systemic toxicity from levothyroxine. The patient had a history of paroxysmal atrial fibrillation on beta-blocker therapy and hypothyroidism and developed symptomatic junctional bradycardia following a lapse in her thyroid supplementation. Upon initiation of hormonal replacement therapy, serial TSH levels were assessed as she continued to have episodic bradycardia with severe hypertension. Given the lack of evidence correlating TSH levels to HR, this case report calls for further studies to be conducted to create reliable guidelines in therapeutic management to prevent bradycardia events while minimizing systemic levothyroxine toxicity.

## Introduction

Cardiovascular and thyroid hormone functions are regulated by intrinsic homeostatic processes. This relationship has been well established by multiple studies, as noted by Klein and Ojamaa [1], which show both low and high levels of thyroid hormone have opposing effects on the cardiovascular system. With elevated thyroid hormone, one would expect a hyperadrenergic state leading to tachycardia, increased oxygen consumption, and increased cardiac workload. Long-term sequelae of these effects could be myocardial hypertrophy with increased left ventricular mass and impaired diastolic filling, which increases the risk of heart failure and stroke. On the contrary, depressed thyroid hormone generally leads to bradycardia with reflexive increased systemic vascular resistance, which decreases stroke volume, thus increasing the risk of heart failure and stroke [1]. Given this important hormonal regulation of the cardiovascular system, it is imperative that thyroid hormone levels be properly managed to maintain a euthyroid state. In this case, we highlight the importance of thyroid hormone regulation in a patient who develops junctional bradycardia during a lapse in thyroid hormone supplementation and the dangerous side effects of excess thyroid hormone supplementation. This case highlights the need for further studies correlating thyroid-stimulating hormone (TSH) levels to heart rate in acutely symptomatic patients to assist in safe hormonal supplementation. This case report was presented as a complex clinical case poster at the Annual Scientific Session of the American College of Cardiology together with the World Congress of Cardiology on March 5, 2023.

## Case presentation

Our patient is a 74-year-old female with a past medical history of paroxysmal atrial fibrillation (pAF), hypertension, and gastric ulcers who presented with left-sided facial droop and left upper extremity weakness for six hours prior to admission. The patient denied similar symptoms in the past and reported compliance with carvedilol, apixiban, and pantoprazole. On presentation, her vital signs were significant for a heart rate of 100 beats per minute, a blood pressure of 110/78 mmHg, a respiratory rate of 19 breaths per minute, and pulse oximetry of 99% in room air. On exam, the patient was noted to have left facial droop with loss of ipsilateral sensation, with ⅖ muscle strength in her left upper extremity, and an irregularly irregular tachycardia on auscultation. The initial electrocardiogram (ECG) showed atrial fibrillation at a heart rate of 100 with a normal axis and probable left ventricular hypertrophy. Initial neuroimaging was significant for an acute ischemic stroke in the right middle cerebral artery territory. Given her history of ulcers and late presentation, she was deemed unsuitable for thrombolytic therapy. Due to large vessel occlusion, she was admitted to the hospital and sent for a thrombectomy, but the patient was found to have a distal clot burden not amenable to intervention, and she was subsequently admitted for further care.

**Figure 1:**
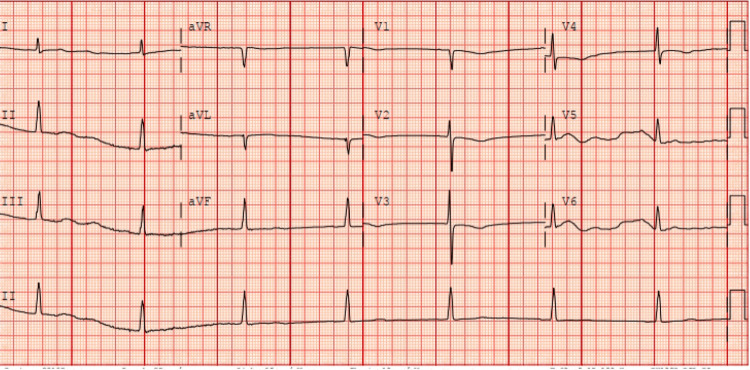
ECG showing junctional bradycardia with a heart rate of 42, absent P waves ECG: electrocardiogram

**Figure 2:**
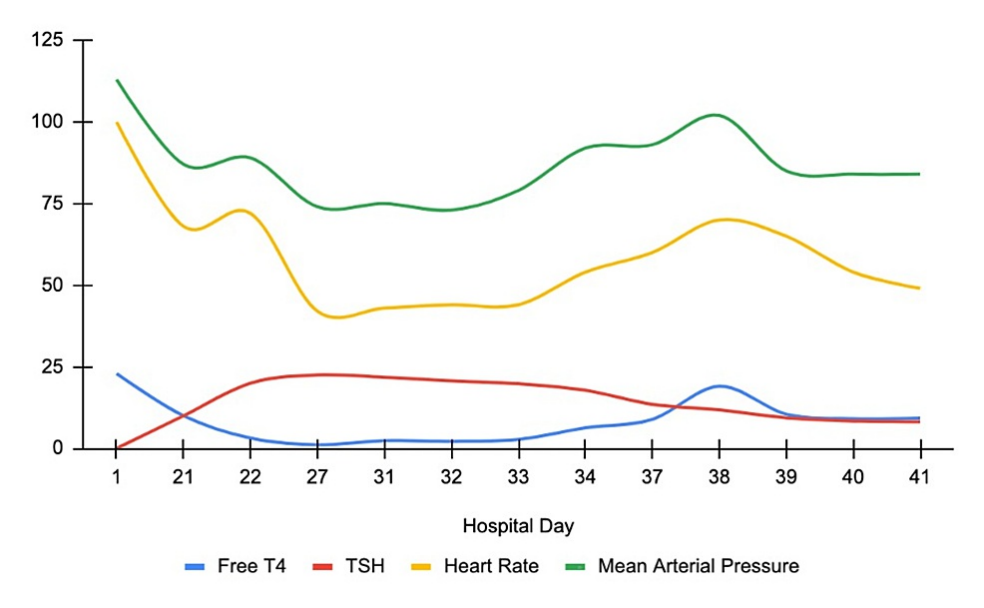
Time vs serial testing Day 1: admission; Day 21: carvedilol cessation with a slight improvement in heart rate; Day 27: IV levothyroxine administration with improved heart rate and increased mean arterial pressure; Day 38: concomitant PO levothyroxine administration; Day 40: only PO levothyroxine with improved heart rate and mean arterial pressure IV: intravenous; PO: per oral

During her hospital stay, the patient was noted to have intermittent atrial fibrillation with a heart rate of less than 90 bpm on home carvedilol. Due to prolonged rehabilitation needs, she remained inpatient, and on hospital day 20, her heart rate began to decrease to 40 to 50 bpm with fluctuating mentation, and her symptoms were thought to be due to either pathologic bradycardia, stroke progression, or acute delirium. The patient was only on carvedilol and pantoprazole. The ECG showed sinus bradycardia with a normal P-R interval and uneventful telemetry. A non-contrast computed tomography (CT) head scan showed petechial hemorrhages around the site of previous ischemia, and the delirium workup was negative, including normal electrolyte levels and no signs of infection. At this time, beta-blocker therapy was discontinued to prevent further bradycardia. Her heart rate and symptoms initially improved, but the heart rate began to decrease three days after beta-blocker cessation. She continued to complain of a headache, and a repeat ECG on hospital day 24 showed junctional bradycardia at 42 beats per minute (bpm) (Figure 1), with otherwise stable vitals and electrolytes. Since the previously seen hemorrhages were likely a natural progression of her ischemic stroke and there were no new focal deficits, other causes for her headache and bradycardia were explored. On initial admission, her TSH was 0.012 U/mL with free thyroxine (T4) of 1.36 ng/dl, but on hospital day 26, she was found to have a TSH level of 22.5 U/mL (normal limits 0.5-5.0 U/mL) with free triiodothyronine (T3) of 0.89 pg/mL (normal limits 2.3-4.1 pg/mL) and free thyroxine (T4) of 0.38 ng/dl (normal limits 0.9-2.3 ng/dl). The decision was made to forgo atropine and dopamine therapy as the bradycardia was thought to be secondary to her thyroid disorder; therefore, the patient was loaded on IV levothyroxine 150 mcg followed by 60 mcg/hr with an improvement of her heart rate to >60 bpm. Further history from the patient revealed that the patient had been on chronic thyroid supplementation at home but did not provide the hospital with this information on admission. She remained inpatient for serial thyroid testing (Figure 2, Table 1) to assess symptom improvement but developed severe hypertension on IV levothyroxine. Given her cerebral hemorrhages, nifedipine and chlorthalidone were required to control her blood pressure to prevent further bleeding as she awaited symptom improvement on the IV levothyroxine therapy. After 10 days of therapy, the patient showed a stable heart rate of >60 bpm with no new neurologic deficits, and she was safely transitioned to oral levothyroxine 75 mcg daily. Once the IV levothyroxine therapy was discontinued, her blood pressure showed significant improvement, and both nifedipine and chlorthalidone were discontinued. The patient’s symptoms resolved with supplementation, and her heart rate improved with an occasional junctional rhythm. The patient was discharged to a rehab facility with instructions to follow up with her primary provider, neurologist, and cardiologist.

**Table 1:** Time vs serial testing Day 1: admission; Day 21: carvedilol cessation; Day 27: IV levothyroxine administration; Day 38: concomitant PO levothyroxine administration; Day 40: only PO levothyroxine administration TSH: thyroid-stimulating hormone; T4: thyroxine

Hospital day	Free T4	TSH	Heart rate	Mean arterial pressure
1	22.93	0.012	100	113
21	10	10	68	87
22	3.16	20	72	89
27	1.12	22.5	42	74
31	2.39	21.8	43	75
32	2.19	20.7	44	73
33	2.75	19.8	44	79
34	6.31	17.8	54	92
37	8.91	13.5	60	93
38	19.05	11.8	70	102
39	10.47	9.4	65	85
40	9.12	8.4	54	84
41	9.33	8.2	49	84

## Discussion

Bradycardia at an electrochemical level is defined by conduction across cardiac myocytes occurring at rates <60 bpm [2]. Although it may be physiologic, there are many pathologic slow rhythms that can lead to a myriad of organ dysfunctions, including renal failure, cerebral ischemia, and pulmonary edema [3]. If a pathologic rhythm is found, the clinical correlation should be investigated to assess the next best step in intervening [4]. Clinical symptoms may be variable, but ones to be vigilant for include syncope, dizziness, confusion, and heart failure.

When bradyarrhythmias arise, one must rule out secondary causes such as infections, medications, hormonal imbalances, abnormal automaticity, and myocardial infarction. In our case, the two major factors causing dysfunction were medications and hormonal imbalance. We ruled out infection due to a lack of fever and source; abnormal automaticity was less likely in this patient with no prior history of bradyarrhythmias and no significant tachycardia that was not explained by a sympathetic response; and we ruled out myocardial infarction given her lack of ECG findings and positive biochemical markers. For her pAF, the patient was taking carvedilol, a beta-blocker that works on the cardiac atrioventricular node to slow conduction, causing sinus bradycardia and possibly heart block in certain patients. Bradycardia induced by beta-blockers is usually transient and may abate with drug cessation [5]. Our patient had only a minor improvement in heart rate after cessation, suggesting another etiology for her bradycardia.

Chemical testing in this case revealed a high TSH level with low T3 and T4 levels, pointing to hypothyroidism as a possible culprit. Abnormal thyroid hormone affects the cardiac system such that low T3 and T4 levels lead to decreased inotropy and chronotropy. These changes can lead to significant cardiac dysfunction and eventual heart failure [1]. To prevent these outcomes, patients are usually supplemented with exogenous thyroid hormone. When supplementing oral thyroid hormone, drug effects can last three to five days. In acute symptomatic settings, the intravenous route is preferred, with a drug effect time of four to six hours [6]. Our patient did show a good chronotropic response with stabilization of symptoms and heart rate after 10 days of IV therapy, but it caused a significant elevation in mean arterial pressure. Given these elevations in pressure and her recent cerebral hemorrhage, multiple anti-hypertensive agents were required to maintain a stable mean arterial pressure. There are no studies that have observed the expected recovery time of heart rate after levothyroxine administration, so there are no clear guidelines on the timeline for recovery. Further research on expected recovery time may benefit clinicians by limiting adverse outcomes.

When thyroid supplementation is given, clinicians must mitigate any potential side effects while awaiting clinical improvement. IV levothyroxine supplementation causes the systemic effects of thyroid hormone to become more pronounced, leading to unwanted side effects such as hypertension. In patients with other comorbidities, these side effects can have detrimental outcomes. Given our patient’s recent ischemic stroke with signs of hemorrhagic conversion, multiple antihypertensives were used to control her blood pressure to prevent the expansion of her intracranial hemorrhage. Although thyroid dosing could have been reduced to reduce the blood pressure effect, the chronotropic effects would have been minimized, diminishing the treatment.

## Conclusions

The use of beta-blocker agents in patients with pathologic tachyarrhythmias is well known. In particular, atrial fibrillation with rapid ventricular rates is controlled with negative chronotropic medications such as beta-blockers. These therapies, although beneficial, may be harmful if not properly titrated within a patient's clinical presentation. As such, patients with underlying pathologic arrhythmias who develop bradycardia when on negative chronotropic medications should have a thorough evaluation of pathologic bradycardia causes. These causes include hormonal abnormalities such as thyroid hormone dysregulation.

Regardless of the wide range of presentations of bradycardia, management focuses on controlling modifiable risk factors while minimizing severe bradycardia events and symptoms. In the case of inadequate thyroid hormone leading to bradycardia, supplementation is recommended to maintain cardiovascular homeostasis. While on thyroid supplementation, other cardiovascular risks can arise, and clinical judgment currently guides dose adjustment. However, further studies should be investigated to correlate TSH levels with heart rate to minimize the systemic toxicity of levothyroxine.

## References

[1] Klein I, Ojamaa K (2001). Thyroid hormone and the cardiovascular system. N Engl J Med.

[2] Hafeez Y, Grossman SA (2023). Sinus Bradycardia. StatPearls [Internet].

[3] Goldschlager N (1988). Underlying assumptions in evaluating "symptomatic bradycardia" (or, are we asking the right questions?). Pacing Clin Electrophysiol.

[4] Hedges JR, Feero S, Shultz B, Easter R, Syverud SA, Dalsey WC (1991). Prehospital transcutaneous cardiac pacing for symptomatic bradycardia. Pacing Clin Electrophysiol.

[5] Khand AU, Rankin AC, Martin W, Taylor J, Gemmell I, Cleland JG (2003). Carvedilol alone or in combination with digoxin for the management of atrial fibrillation in patients with heart failure?. J Am Coll Cardiol.

[6] von Hafe M, Neves JS, Vale C, Borges-Canha M, Leite-Moreira A (2019). The impact of thyroid hormone dysfunction on ischemic heart disease. Endocr Connect.

